# A descriptive study of ciguatera fish poisoning in Cook Islands dogs and cats: Exposure history, clinical signs, and formulation of a case definition

**DOI:** 10.14202/vetworld.2020.372-385

**Published:** 2020-02-27

**Authors:** Michelle J. Gray, M. Carolyn Gates

**Affiliations:** 1 Master of Veterinary Medicine Program, School of Veterinary Science, Massey University, Palmerston North, New Zealand; 2 EpiCentre, School of Veterinary Science, Massey University, Palmerston North, New Zealand

**Keywords:** case definition, cats, ciguatera, clinical signs, Cook Islands, dogs, exposure

## Abstract

**Background and Aim:**

Ciguatera fish poisoning (CFP) is a multisystem toxicosis caused by the ingestion of warm water marine species. Dogs and cats are susceptible to CFP, but there is little published and much unknown about the condition in these species. This study aims to describe the syndrome of CFP in dogs and cats and to develop a case definition.

**Materials and Methods:**

Six years (March 2011-February 2017) of medical records from the Esther Honey Foundation Animal Clinic (the only veterinary clinic in the Cook Islands during the study period) were reviewed to identify cases of CFP. Data relating to exposure history and clinical signs were collected.

**Results:**

Two hundred forty-six cases of CFP were identified, comprising 165 dogs and 81 cats. Fish ingestion was documented in 29% of cases. Reef/lagoon fish and moray eels were most commonly implicated. The toxicosis was characterized by motor dysfunction with a high frequency of ataxia and paresis/paralysis/recumbency. Respiratory and gastrointestinal systems were also affected, especially in canine CFP cases. A multi-tiered case definition and a diagnostic algorithm for CFP in dogs and cats were developed based upon the findings of this study and a review of the existing literature.

**Conclusion:**

This case series is the largest study of canine and feline CFP to date. It documents the exposure history of cases and describes in detail clinical signs of the toxicosis. It also proposes a system of case classification that has the potential to both assist the diagnosis of CFP and facilitate future surveillance and research activities.

## Introduction

Ciguatera fish poisoning (CFP) is a multisystem toxicosis resulting from the ingestion of fish containing ciguatoxins (CTXs). Experimental studies have proven that dogs and cats are susceptible to CFP [1-6]. However, little or no research has been conducted into canine and feline CFP in the past 30 years. Publications have been limited to case reports [7-12] and general reviews of the toxicity [13-19].

CTXs are the fundamental cause of CFP [20]. CTXs are formed through oxidation of gambiertoxins, which are produced by certain strains of *Gambierdiscus* spp. microalgae [21-24]. Herbivorous fish species bioaccumulate CTXs when they ingest toxic *Gambierdiscus* spp. [25], and carnivorous fish become toxic when they ingest CTX containing herbivores [26]. The estimated half-life of CTX in fish is 264 days [22]. Toxic fish still look, smell and taste normal, and CTXs are unaffected by freezing, cooking, and drying [23]. CFP occurs when susceptible species ingest CTX containing fish. Table-1 [1,3,5-8,10-12] details the fish species documented to cause CFP in dogs and cats.

**Table-1:** Species of fish documented to cause ciguatera fish poisoning in dogs and cats.

Family	Species	Common name	Field cases	Experimental disease
Snappers (*Lutjanidae*)	*Lutjanus bohar*	Red Snapper	2 reports [10,11]	Yes [1,3]
	*Lutjanus miniatus*	Red throat emperor		Yes [1]
	*Aprion virescens*	Green jobfish		Yes [1]
Groupers (*Serranidae*)	*Cephalopholis argus*	Peacock grouper		Yes [1]
	*Epinephelus microdon*	Camouflage grouper		Yes [1]
	*Plectropomus leopardus*	Coral trout	1 report [7]	Yes [1]
Moray eels (*Muraenidae*)	*Gymnothorax javanicus*	Giant moray		Yes [5,6]
Parrotfish (*Scaridae*)	*Scarus gibbus* (now *Chlorurus gibbus*)	Heavy-beak parrotfish		Yes [6]
	*Scarus jonesi* (now *Chlorurus frontalis*)	Tan-faced parrotfish		Yes [1]
Mackerel/tuna (*Scombridae*)	*Scomberomorus commerson*	Narrow-barred Spanish mackerel	2 reports [8,12]	
	*Gymnosarda unicolor*	Dogtooth tuna		Yes [1]
Jacks (*Carangidae*)	*Caranx melampygus*	Bluefin trevally		Yes [1]
Barracuda (*Sphyraenidae*)	*Sphyraena barracuda*	Great barracuda		Yes [1]
Unicornfish (*Acanthuridae*)	*Naso unicornis*	Bluespine unicornfish		Yes [1]
Wrasse (*Labridae*)	*Cheilinus undulatus*	Humphead Maori wrasse		Yes [1]

CTXs cause dysfunction of excitable cells. They bind with high affinity to site five of voltage-sensitive sodium channels, causing them to aberrantly open at resting membrane potential [23]. The resulting intracellular flow of sodium causes spontaneous and repetitive action potentials [27,28]. CTXs are extremely potent with toxicity observed at doses as low as 48 pg/kg bodyweight [29].

The clinical presentation of CFP can differ between species [30]. Human CFP generally manifests within 12-24 h of fish ingestion and is characterized by a combination of gastrointestinal and sensory abnormalities [31-34]. The time to onset of clinical signs in dogs and cats is comparable (Table-2), but the symptomology appears distinct. The most consistently reported abnormalities in canine and feline CFP are ataxia and paresis, often beginning in the hindquarters before affecting all four limbs [2,4]. Other signs reported in dogs and cats but rare in human CFP include opisthotonos, tremors, convulsions, nystagmus, and groaning [7,15]. The clinical signs of canine and feline CFP as reported in the literature are summarized in Table-3 [1-15,17-19].

**Table-2:** Time to onset of illness reported for ciguatera fish poisoning in dogs and cats.

Species	Reference	Type of publication	Time to onset of illness
Dogs	Kawakubo and Kikuchi [4]	Experimental study	1-6 h
	Anonymous [7]	Case report/series	1 day
Cats	Bagnis and Fevai [2]	Experimental study	1-10 h
	Clark and Whitwell [8]	Case report/series	6 h
	Kemppainen *et al.* [9]	Case report/series	A few hours
	Tonge *et al.* [12]	Case report/series	18 h
	Lewis [17]	Review/general article	>3 h
	McPherson [18]	Review/general article	3-6 h
	Seawright [19]	Review/general article	<6 h

**Table-3:** Clinical signs reported for ciguatera fish poisoning in dogs and cats.

Species	Dogs						Cats													
		
Reference	[4]	[7]	[11]	[13]	[14]	[15]	[1]	[2]	[3]	[5]	[6]	[8]	[9]	[10]	[12]	[14]	[15]	[17]	[18]	[19]
Type of report^1^	E	C	C	R	R	R	E	E	E	E	E	C	C	C	C	R	R	R	R	R
Neurologic signs																				
Paresis/paralysis	X			X	X	X	X	X	X		X	X	X	X	X	X	X	X	X	X
Ataxia		X	X	X	X	X	X	X	X		X	X			X	X	X	X	X	X
Recumbency	X			X			X	X	X				X							
Convulsions/seizures		X				X		X									X			
Lethargy			X			X		X									X			
Lacrimation								X								X		X	X	
Rigidity/opisthotonos		X				X											X			
Tremors					X	X		X									X			
Hyperesthesia								X					X							
Mydriasis	X							X												
Nystagmus					X	X											X			
Cardiovascular and respiratory signs																				
Dyspnea	X									X	X				X	X		X	X	X
Tachycardia								X		X	X		X						X	
Bradycardia								X		X	X								X	
Arrhythmias								X								X		X	X	
Groaning		X		X				X												
Hypotension										X								X	X	
Gastrointestinal signs																				
Vomiting			X		X	X			X		X				X	X	X	X	X	X
Diarrhea					X	X		X	X		X				X		X	X		X
Salivation	X							X	X			X			X	X		X	X	X
Inappetence/anorexia				X								X							X	X
Abdominal pain						X											X			

1 C= clinical report or case series; E= experimental study; R= review of the toxicity

There is currently no diagnostic test for CFP [23]. Cases (both human and animal) are diagnosed presumptively based on history and clinical presentation. Case definitions have been developed for human CFP [35-37], but to date, there has been no attempt to define diagnostic criteria for canine or feline CFP. “Gold standard” diagnosis of CFP includes detection of CTX in the implicated fish [23]. The testing of fish is unfortunately costly and requires specialized laboratory equipment [38,39]. None of the published case reports of CFP in dogs and cats have included laboratory confirmation.

Documenting the clinical presentation of CFP in dogs and cats is important given that CFP is currently diagnosed by pattern recognition. The existing evidence base is limited – either dated and experimental or derived from short case reports. For veterinarians to accurately identify CFP cases, the symptomology must be well described. A case definition (ideally one based on the symptomology of a large number of cases) would facilitate standardized CFP diagnosis for clinicians and also provide researchers with a means of distinguishing cases from non-cases.

This study is the second in a series describing CFP in dogs and cats in the Cook Islands. This study aimed to document the exposure history and symptomology of CFP cases and to use this information to develop a clinical case definition for CFP in dogs and cats.

## Materials and Methods

### Ethical approval

This retrospective review of case records was deemed to not require ethics approval (Massey University).

### Data collection

The location and methodology of this study, along with the findings regarding the demographic, temporal, and spatial distribution of cases, are described in detail elsewhere [40]. In brief, the medical records of the Esther Honey Foundation (EHF) Animal Clinic, Rarotonga, were searched for cases with a presumptive diagnosis of CFP. Cases presenting in the 6-year period March 2011-February 2017 were considered for inclusion. Eligible patient files were searched for the variables of interest (Supplementary Table-1): Details of toxin exposure and clinical signs. Data were collated using Epi-Info software (version 7.2.1.0, CDC, Atlanta, USA).

### Statistical analysis

Time to onset of illness was calculated as the date of onset of clinical signs – date of fish ingestion. Descriptive statistics were performed in Epi-Info.

To explore the possibility that animals without known exposure to fish were mistakenly diagnosed as having CFP, cases were grouped by species and exposure history. Univariate analyses were then performed to identify any differences in symptomology. Specifically, Fisher’s exact test was used to evaluate the association between known/unknown exposure history and the presence of each of 25 individual clinical signs. Associations with p<0.05 were considered statistically significant. The same procedure was used to compare differences in clinical signs between canine and feline cases of CFP to evaluate whether the clinical presentation differs between species.

A rudimentary assessment of the timing/evolution of clinical signs of CFP was made by comparing the proportion of clinical signs manifest on presentation with the proportion of cases affected in total.

To develop a case definition for canine and feline CFP, existing case definitions for human CFP [35-37] were used as a foundation. Exposure and clinical criteria were modified to reflect the results of this study, and previous reports of canine and feline CFP. The diagnostic criteria were integrated into a case definition matrix to allow for differing levels of diagnostic certainty. A step-wise algorithm for the case definition was then created.

## Results

Two hundred and forty-six cases with a presumptive diagnosis of CFP were identified from the 6-year pool of medical records. These comprised of 165 dogs and 81 cats.

### Exposure

Fish ingestion before the illness was documented in 71 cases (28.9%). The animals with known fish exposure included 48 dogs (29.1%) and 23 cats (28.4%).

The type of fish was noted in 23 cases (31.0% of those with known fish ingestion). Reef/lagoon fish and moray eels were most commonly implicated (Table-4).

**Table-4:** Species of fish involved in ciguatera fish poisoning cases.

Fish	Cases	Percentage	Notes
Not specified	49	69.0	Includes 5 cases where the owner shared the fish and developed CFP, and 7 cases where fish was scavenged from the beach
Reef/ lagoon	9	12.7	Species not identified. Includes a case which also ate moray eel*
Moray eel	7	9.9	Includes a case which also ate unspecified lagoon fish*
Tuna	3	4.2	Includes 2 cases recorded as “possibly tuna”
Mackerel	1	1.4	
Parrot-fish	1	1.4	
Trevally	1	1.4	
Blue starfish	1	1.4	
Total	72	101.4*	*1 case ingested both lagoon fish and moray eel

CFP=Ciguatera fish poisoning

The source of exposure was noted in 44 cases (62.0% of those with known fish ingestion). Dogs most commonly scavenged fish, while cats were more often fed fish by the owner (Table-5). The source of exposure in dogs and cats was significantly different, based on Fisher’s exact test (p=0.0196).

**Table-5:** Source of fish ingested by ciguatera fish poisoning cases.

Source of fish	Canine cases (%)		Feline cases (%)		Combined cases (%)	
Fed by owner	8	(16.7)	13	(56.5)	21	(29.6)
Scavenged	15	(31.3)	4	(17.4)	19	(26.8)
Neighbor had fish	3	(6.3)	1	(4.3)	4	(5.6)
Unknown	22	(45.8)	5	(21.7)	27	(38.0)
Total	48	(100)	23	(100)	71	(100)

The date of exposure was recorded in 39 cases (54.9% of those with known fish ingestion). The mean time to onset (from ingestion to observation of clinical signs) for these animals was 0.9 days (Table-6).

**Table-6:** Time to onset of ciguatera fish poisoning cases.

Onset of clinical signs	Canine cases (%)		Feline cases (%)		Combined cases (%)	
Same day as ingestion	9	(31.0)	1	(10.0)	10	(25.6)
1 day following ingestion	19	(65.5)	6	(60.0)	25	(64.1)
2 days following ingestion	1^1^	(3.4)	1	(10.0)	2	(5.1)
3 days following ingestion	0	(0.0)	2^2^	(20.0)	2	(5.1)
Total	29	(100)	10	(100)	39	(100)
Mean time to onset (days)	0.72		1.40		0.90	

1 One dog reported to ingest fish either 1 or 2 days prior to illness,

2 One cat missing for 2 days between ingestion and presentation

Nine animals had more than one documented episode of CFP. Six dogs and three cats suffered a total of 19 episodes of CFP. Fish ingestion was known or suspected in seven of the nine animals (77.8%) and in 11 of the 19 episodes (57.9%) (Table-7).

**Table-7:** Animals with successive episodes of ciguatera fish poisoning.

Species	Number of CFP episodes	Date of episodes	Known fish ingestion
Cat	3	August 7, 2012	Yes
		September 8, 2013	Yes
		May 20, 2016	Yes
Cat	2	April 10, 2012	No
		June 12, 2014	Yes
Cat	2	January 7, 2013	No
		April 18, 2013	Yes
Dog	2	December 27, 2011	Yes
		October 24, 2012	Yes
Dog	2	November 29, 2012	No
		February 1, 2013	No
Dog	2	June 18, 2012	Yes
		September 22, 2012	Yes
Dog	2	May 3, 2011	No
		June 4, 2011	No
Dog	2	August 8, 2015	Yes
		March 13, 2016	No
Dog	2	August 8, 2015	Yes
		March 14, 2016	No

CFP=Ciguatera fish poisoning

### Clinical signs

Two hundred and thirty-eight medical records (96.7% of all cases) contained relevant data. Files for the remaining eight cases (four dogs and four cats) failed to document any clinical signs.

The case files documented many previously undescribed clinical signs of CFP in dogs and cats. Those that affected >5 cases included hypothermia (n=37); agitation/restlessness (n=16); reduced patella reflex (n=13); skin lesions/pressure sores (n=11); hemorrhagic diarrhea (n=10); non-specific pain (n=8); poor body condition (n=8); ocular discharge (n=8); third eyelid protrusion (n=7); thrashing/erratic movements (n=7); decreased withdrawal response (n=7); and reduced menace response (n=6).

Also previously undescribed are the effects of CFP in gravid dogs and cats. Four cases (two dogs and two cats) were pregnant. Abortion, stillbirth, poor neonatal viability, and maternal complications were observed. Details are provided in Table-8.

**Table-8:** Outcome of ciguatera fish poisoning in pregnant animals.

Species	Details of pregnancy	Outcome for mother
Dog	Vulval discharge noted during hospitalization (treated with antibiotics). Pregnancy diagnosed when spayed after recovery (stage of gestation unspecified)	Recovered in 21 days
Dog	Aborted 2 mid-term puppies on day 17 of hospitalization	Recovered in 32 days
Cat	Assessed as mid-stage pregnant on presentation	Died on day 3, respiratory arrest after suspected aspiration
Cat	Gave birth on day 3 hospitalization – 2 live kittens, 1 stillborn. Mother had no milk, kittens died within 24 h despite attempts to foster	Recovered in 22 days

Regarding the clinical signs previously documented in the literature (Table-3), all except for hypotension, lacrimation, and mydriasis were observed in the study population. Table-9 lists the overall frequency of previously documented clinical signs in this case series.

**Table-9:** Frequency of clinical signs observed in ciguatera fish poisoning cases.

Clinical signs	Number of reports	Percent^1^
Ataxia	164	68.9
Recumbency	147	61.8
Inappetence/anorexia	133	55.9
Paresis/paralysis/weakness	116	48.7
Hypertonia/extensor rigidity	112	47.1
Tachypnea/dyspnea	109	45.8
Unable to walk	79	33.2
Hindlimbs worse than forelimbs	66	27.7
Opisthotonos	65	27.3
Obtunded mentation	63	26.5
Groaning	62	26.1
Vocalization	60	25.2
Tremors	59	24.8
Nystagmus	51	21.4
Dehydration	46	19.3
Hypersalivation	44	18.5
Proprioceptive deficits	44	18.5
Lethargy	34	14.3
Vomiting	27	11.3
Diarrhea	27	11.3
Cardiac irregularities^2^	23	9.7
Hyperesthesia/dysesthesia	20	8.4
Convulsions/seizures	19	8.0
Abdominal discomfort	16	6.7
No gag reflex	16	6.7
Hypotension	0	0.0
Lacrimation	0	0.0
Mydriasis	0	0.0

1 Of n=238 cases in which one or more clinical signs were documented,

2 Includes 14 incidences of bradycardia and 6 of tachycardia

Comparing the frequency of clinical signs documented in dogs with known fish ingestion versus dogs with unknown exposure history, only three (of 25) clinical signs were associated (p<0.05) with exposure history (Supplementary Table-2). Dogs with a known history of fish ingestion were more likely to have diarrhea (odds ratio [OR] = 2.9, 95% confidence interval [CI] 1.1-7.8) and cardiac irregularities (OR=3.1, 95% CI 1.0-10.0), while abdominal pain was only recorded in cases with unknown exposure history (OR=0, 95% CI 0.0-0.6).

A comparison of the frequency of clinical signs reported from cats with documented fish ingestion versus cats with unknown exposure history did not identify any significant (p<0.05) differences (Supplementary Table-3).

Comparing the frequency of clinical signs reported in dogs versus cats, there were seven significant (p<0.05) differences between the species (Supplementary Table-4). Specifically, there was a higher frequency of dyspnea (OR=0.3, 95% CI 0.2-0.6); groaning (OR=0.1, 95% CI 0.0-0.3); vomiting (OR=0.2, 95% CI 0.0-0.8); diarrhea (OR=0.2, 95% CI 0.0-0.8); and abdominal discomfort (OR=0.1, 95% CI 0.0-0.8) in dogs. Cats had higher rates of dehydration (OR=3.8, 95% CI 1.9-7.9) and dysesthesia/hyperesthesia (OR=4.1, 95% CI=1.5-11.8).

A comparison of the initial clinical presentation of cases with the clinical signs observed through the entirety of hospitalization found that abdominal discomfort, cardiac irregularities, and tachypnea/dyspnea tended to manifest early (abnormality noted on presentation in 100%, 83%, and 82% of occurrences, respectively). In contrast, convulsions/seizures, opisthotonos, and nystagmus were comparatively rare on presentation (noted on presentation in 26%, 31%, and 37% of occurrences) (Supplementary Table-5).

### Case definition

**Figure-1:**
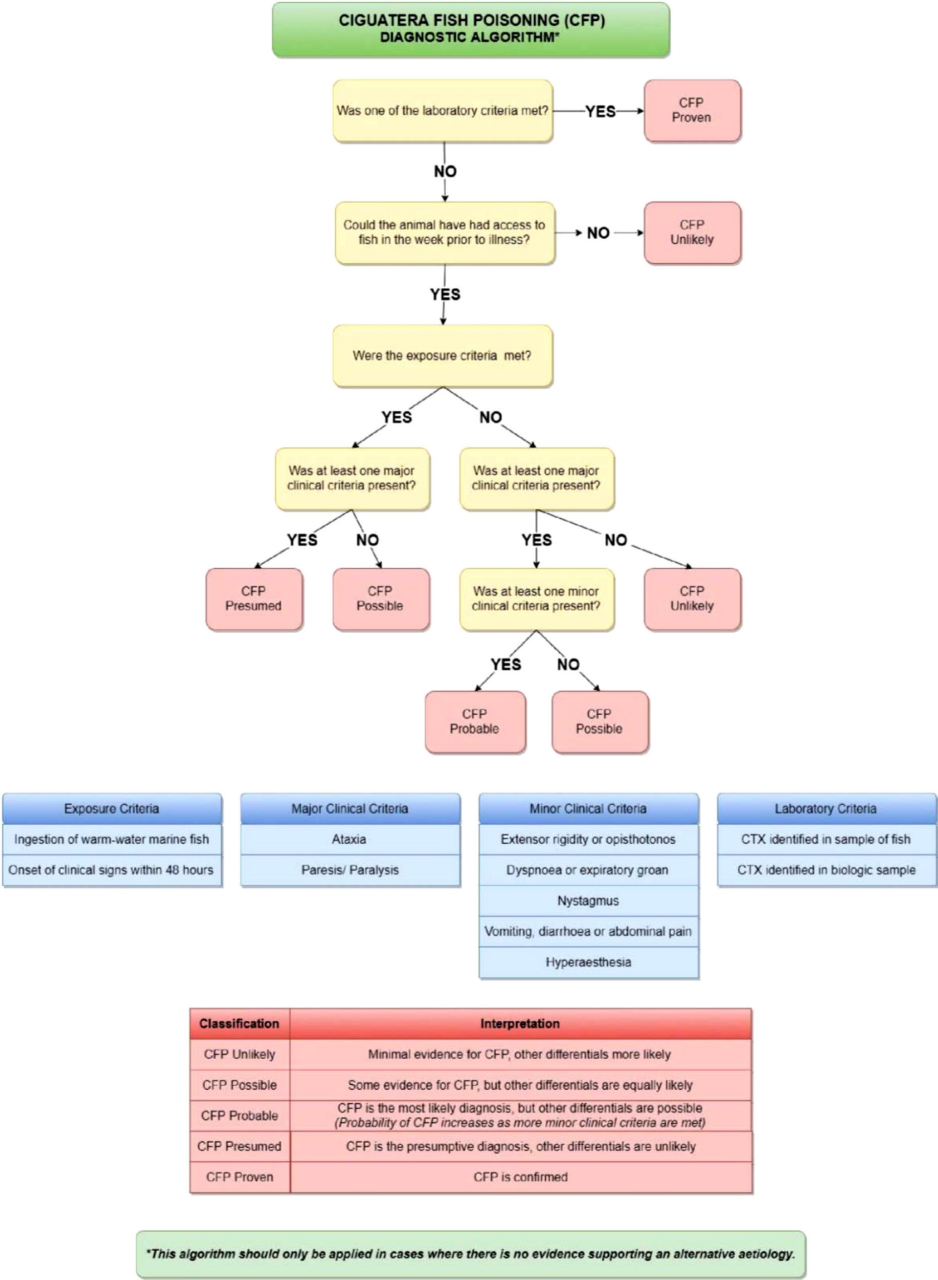
Diagnostic algorithm for ciguatera fish poisoning based on the proposed case definition.

Table-10 presents the proposed multi-level case definition for canine and feline CFP. Table-11 details the exposure, clinical, and laboratory criteria used to determine the level of diagnostic certainty. A step-wise diagnostic algorithm for the proposed case definition is presented in Figure-1.

**Table-10:** Proposed case definition for ciguatera fish poisoning in dogs and cats.

Classification	Interpretation	Minimum requirement
Proven	CFP is the confirmed diagnosis	Meets one or more laboratory criteria
Presumed	CFP is the presumptive diagnosis; other differentials are unlikely	Meets both exposure criteria, AND one or more major clinical criteria
Probable	CFP is the most likely diagnosis; but other differentials are possible	Meets one or more major clinical criteria, AND one or more minor clinical criteria
Possible	There is some evidence for CFP; but other differentials are equally likely	Meets both exposure criteria, OR one or more major clinical criteria
Unlikely	There is minimal evidence for CFP; other differentials are more likely	Does not meet any of the above requirements

CFP=Ciguatera fish poisoning

**Table-11:** Proposed criteria for a case definition for ciguatera fish poisoning in dogs and cats.

Category	Criteria
Exposure criteria	Ingestion of warm-water marine species
	Onset of clinical signs within 48 h of ingestion
Major clinical criteria	Ataxia
	Paresis or paralysis
Minor clinical criteria	Extensor rigidity or opisthotonos
	Dyspnea or expiratory groan
	Nystagmus
	Vomiting, diarrhea, or abdominal pain
	Hyperesthesia
Laboratory criteria	CTX identification in sample of ingested fish
	CTX identification in the biologic sample from case

CTX=Ciguatoxins

Under the proposed case definition, 65% of the current case series had either presumed or probable CFP (Table-12). Excluding files that lacked clinical signs, and assuming cases with recumbency were paretic, increases the proportion of presumed/probable cases to 79%. Classifying cases based solely on the signs recorded on day 1 of hospitalization reduces the proportion of presumed/probable cases to 39%.

**Table-12:** Classification of the study population under the proposed case definition for ciguatera fish poisoning in dogs and cats.

Study population	Number of cases	Classification (%)			

		Presumed CFP	Probable CFP	Possible CFP	Unlikely CFP
Dogs	165	12	54	15	19
Cats	81	9	53	21	17
All cases	246	11	54	17	18
Cases with recorded symptoms	238	11	55	17	16
All cases (recumbency assumed to reflect paresis/paralysis)	246	12	64	17	7
Cases with recorded symptoms (recumbency assumed to reflect paresis/paralysis)	238	13	66	18	4
All cases (classification on day 1 of hospitalization)	246	10	29	24	37

CFP=Ciguatera fish poisoning

## Discussion

### Study limitations

There are limitations inherent in the methodology of this study that should be considered when interpreting the results. Classification of cases was based solely on the attending clinician’s opinion. Missed diagnoses and misdiagnoses are both possible and may affect the clinical picture detailed. The quality of the clinical records varied and some clinical signs probably went unrecorded. Consequently, the true frequency of clinical signs is likely higher than that reported in the results. Regarding the statistical analyses, the small size of certain subgroups means results may be impacted by random error. The large number of individual statistical tests performed means that false findings from type 1 error are also possible.

### Exposure

In this study, the ingestion of fish was documented in less than a third of CFP cases. Fish exposure was more frequently documented in animals who suffered repeated episodes of CFP. This may be because animals who frequently catch or scavenge fish are both more likely to be observed and more likely to suffer CFP. Another contributing factor could be increased owner awareness and observation after the first episode of CFP. In one case, it was because the owner continued feeding lagoon fish despite knowing the risk.

The species of fish associated with ciguatera in this study accord with those identified in previous publications. Moray eels, which were most frequently specified, are known to be highly ciguateric [41]. Tuna, mackerel, parrot-fish, and trevally have all been reported to cause human CFP in the Cook Islands [42]. One anomaly was a case associated with the ingestion of blue starfish tentacles (probable species *Linckia laevigata*). Starfish have not previously been associated with clinical CFP, although other invertebrates have [42]. The potential for starfish to act as a vector of CFP is supported by the research of Silva *et al*. [43] who recently identified CTX in two different starfish species.

In this study, dogs and cats differed in the source of the fish they ingested. Dogs most commonly scavenged fish. This may be attributed to greater beach access, for while both species are commonly free-roaming in the Cook Islands, cats rarely venture down to the water. Cats meanwhile were more often fed fish by their owners. This perhaps reflects owner perceptions that fish are a natural food source for cats. The differences were statistically significant and have implications for disease prevention, suggesting that species-specific education programs may be required.

The time to onset of illness in this study is consistent with that previously reported (Table-2). The average duration of 0.9 days (21.6 h) should not be considered precise, however, as the variable only allowed for whole-day values. The results probably also overestimate the time to onset of illness, because there is inevitably a delay between the onset and observation of clinical signs.

### Clinical signs

In this study, CFP was characterized by locomotor abnormalities, with respiratory and gastrointestinal dysfunction contributing to morbidity. The results are largely consistent with previous reports of the toxicosis. There were, however, differences: Some novel clinical signs were recorded, and a small number of previously reported clinical signs were not identified in the study population.

Hypothermia was documented in 15% of dogs and cats in this study. Given the warm tropical environment, it implies significant thermoregulatory dysfunction. While hypothermia is a recognized feature of CFP in mice [44], it has not been previously reported in dogs or cats. Few articles mention patient temperatures at all, except Bagnis and Fevai [2] who explicitly stated there was no disturbance of thermoregulation in feline CFP. The reason for this discrepancy is unknown.

Hemorrhagic diarrhea was another novel and unexpected clinical sign, observed in ten of the CFP cases (nine dogs and one cat). While diarrhea has often been observed in canine and feline CFP cases (Table-3), the only descriptors ever applied were “profuse” and “watery” [2]. Hemorrhagic diarrhea could be the result of intestinal parasitism. Worms were documented in two of the affected animals (fecal testing not performed in the other eight), and it is conceivable that in a gastrointestinal tract compromised by CFP, worms would induce greater pathology and hemorrhage. Alternatively, it is possible that CFP causes hemorrhagic diarrhea in a small percentage of dogs. Previous canine case reports have been limited in size and may have missed an uncommon clinical sign.

Non-specific pain was noted in eight of the current case series. This contrasts with previous reports, which have only documented abdominal discomfort [15]. It is possible that animals in previous studies did experience non-specific pain, but the clinical sign was considered too subjective to report. Arthralgia, myalgia, and headaches have all been reported in human CFP [45] and any of these could present as non-specific pain in an animal.

A variety of neurologic signs were explicitly documented for the first time in this study. These include agitation/restlessness, third eyelid protrusion, reduced patella and withdrawal reflexes, and reduced menace response. These neurologic abnormalities may have occurred in previous CFP cases but been omitted from publications due to a focus on more overt signs such as ataxia and paralysis.

A final novel discovery was the case files documenting CFP in pregnant dogs and cats. These are significant given that there have been no previous reports of CFP in gravid animals. The effect of CFP in pregnancy is poorly understood even in humans. CFP has been reported to cause a transient increase in fetal movements [46-48]. There have also been isolated reports of abortion [47,49]. In this study, abortion, stillbirth, poor neonatal viability, and maternal complications were observed. While the number of cases was small, and the effect of CFP is likely to depend on the stage of gestation, the findings of this study suggest that the prognosis for canine and feline fetuses exposed to CTX is guarded.

Regarding previously reported clinical signs, hypotension, mydriasis, and lacrimation were not identified in the study population^1^. As the EHF Animal Clinic lacked blood pressure monitoring equipment, no inference can be made from the absence of recorded hypotension. As for mydriasis and lacrimation, these may have been present but not observed or documented in case files. Alternatively, these signs may not occur in naturally occurring CFP, as they have only previously been reported in experimental studies [2,4].

Overall, the symptomology of canine CFP cases with and without known fish ingestion was found to be very similar. There were no significant differences detected in the ten most frequently identified clinical signs (Supplementary Table-2). Only the frequency of diarrhea, cardiac irregularities, and abdominal discomfort differed between the groups. These differences could be due to type 1 error; differences in the speed of presentation (in human CFP gastrointestinal and cardiovascular signs occur early in the course of the disease) [23]; or could represent true differences between the groups. Given the overall similarity in symptomology between the two groups, it is considered that the majority of dogs without documented fish ingestion were correctly diagnosed as having CFP.

The comparison of cats with known fish ingestion versus those with unknown exposure history detected no significant differences in the frequency of clinical signs (Supplementary Table-3). This suggests that cats without documented fish ingestion were correctly diagnosed as having CFP.

Seven significant differences were detected in the comparison of clinical signs between dogs and cats. Dogs exhibited more respiratory and gastrointestinal dysfunction, while dysesthesia/hyperesthesia and dehydration were more common in cats (Supplementary Table-4). Some of the detected differences are not unexpected. Dysesthesia/hyperesthesia has only been reported previously in cats (not dogs) with CFP (Table-3). And cats, being smaller animals, are logically at greater risk of dehydration. Differences in the frequency of respiratory and gastrointestinal signs are, however, an unexpected finding. The results are unlikely to be due to type 1 error given that (with the exception of abdominal discomfort) the p-values were <0.01, and several were exponentially smaller (Supplementary Table-4). It is therefore concluded that differences exist in the symptomology of CFP and dogs and cats.

The timing/evolution of clinical signs in the study population was crudely assessed by comparing signs evident on presentation with those documented at any stage (Supplementary Table-5). In human CFP, cardiovascular and gastrointestinal signs are generally observed early in the course of the disease. In this study, a parallel was seen with cardiac irregularities and abdominal discomfort tending to manifest early, although the same pattern did not hold true for vomiting and diarrhea. The results also showed that convulsions/seizures, opisthotonos, and nystagmus were comparatively rare on presentation in both dogs and cats. This may indicate they develop later in the course of the disease. Alternatively, it may be because these signs occur only intermittently and are difficult to observe without a period of hospitalization.

### Case definition

The number of cases reviewed in this study is considered sufficient to inform the development of a case definition. Neville and Warren [37] used 149 cases as the basis of their case definition for CFP in humans. The number of dogs in this study (165) compares favorably. The data from feline cases (n=81) are less robust but still considered adequate to identify the key clinical features of CFP in cats.

In keeping with the existing case definitions for human CFP [35-37], the proposed case definition uses a combination of exposure, clinical, and laboratory criteria to identify cases (Table-11). However, instead of a binary case/non-case classification, a multi-tiered model is suggested to recognize different levels of diagnostic certainty (Table-10). This is similar to the approach Thundiyil *et al*. and the Brighton Collaboration [50,51]. A diagnostic algorithm was developed for the proposed case definition (Figure-1) based on evidence suggesting this format is more efficient than case definition tables or matrices when determining the level of diagnostic certainty [52].

Exposure criteria (Table-11) establish if there is a temporal link between illness and ingestion of a potentially ciguateric fish. The criterion requiring the onset of clinical signs within 48 h is based on the results of this study and the existing literature. The time frame could be restricted to 24 h, and 90% of animals in this study with known fish ingestion would still meet the criterion. However, given that an animal’s clinical signs could go unobserved for a time, a conservative approach that maximizes case inclusion was deemed appropriate. Case definitions for human CFP require that cases fulfill exposure criteria [35-37]. On the basis that fish ingestion occurred unobserved in two-thirds of the CFP cases in this study, exposure criteria are not mandatory for CFP diagnosis under the proposed case definition, however, meeting the exposure criteria results in a higher level of diagnostic certainty.

No single clinical sign was found to be pathognomonic for CFP in this study. Diagnosis of CFP in dogs and cats, therefore, requires a combination of clinical signs. This contrasts with the human situation where certain characteristic paresthesias/dysesthesias are used as the basis of case definitions [23,37,53]. The proposed clinical criteria (Table-11) aim to achieve a balance of sensitivity and specificity through the application of major and minor categories. Major clinical criteria provide sensitivity and capture the majority of CFP cases. Minor clinical criteria are supporting signs that increase diagnostic specificity.

The proposed major clinical criteria are ataxia and paresis/paralysis. These signs, when combined, identify 78% of dogs and 86% of cats in the current case series. If all recumbent animals were assumed to have paresis/paralysis, the sensitivity increases to 92% and 98%, respectively. The only other clinical signs of comparable frequency were anorexia and recumbency, both of which were considered insufficiently specific to aid diagnosis. The existing literature supports the use of ataxia and paresis/paralysis as indicators of CFP in dogs and cats (Table-3). Of particular note, in both canine and feline bioassays of ciguatera, the outcome was based on the presence and severity of ataxia and paresis/paralysis [1,2,4].

Proposed minor clinical criteria (Table-11) include selected neurologic, respiratory, and gastrointestinal abnormalities drawn from the results of this study. Inclusion was based on their ability to differentiate CFP from other causes of ataxia and paresis/paralysis. The more minor criteria that are met, the greater the probability of CFP. The proposed criteria are consistent with the available literature, in which respiratory and gastrointestinal dysfunction commonly accompany neurologic signs (Table-3).

Laboratory criteria are included in the proposed case definition in the hope that laboratory testing of fish or biologic samples will eventually become a reality to confirm field cases of CFP. To date, only experimental cases would meet these criteria.

Under the proposed case definition, two-thirds of the current case series had either presumed or probable CFP (Table-12). It is likely that more cases would have achieved a high classification had the case definition been applied at the time of hospitalization. The sensitivity of retrospective classification is proportional to the detail of the clinical records, and the clinical detail of the case files in this study ranged from excellent to extremely limited.

The proposed case definition is considered relevant to both dogs and cats. The exposure, laboratory, and major clinical criteria pertain equally to both species. Of the minor clinical criteria, dogs are more likely to have respiratory and gastrointestinal dysfunction, and cats are more likely to exhibit dysesthesia/hyperesthesia (Supplementary Table-4). However, this is accounted for under the structure of the proposed case definition, where the effect on classification is the same regardless, of which (or how many) minor clinical criterion is met.

Potential applications for the proposed case definition include clinical case diagnosis, disease surveillance, and research. For veterinarians unfamiliar with CFP, the classification system indicates the probability of CFP based on simple historical and clinical factors. For disease surveillance, where diagnostic sensitivity is a priority, assessments of CFP incidence could incorporate all cases with a reasonable probability of CFP (i.e. probable, presumed, and proven classifications). For research scenarios where the accidental inclusion of non-cases would adversely affect results (e.g. investigation of risk factors or therapeutic interventions), cases with the highest level of diagnostic certainty (proven and/or presumed classifications) could be selected.

The proposed case definition may not be applicable or accurate under certain circumstances. For example, if there is no possibility of fish exposure in the week preceding illness, or if there is historical or clinical evidence to support a different diagnosis (e.g. tick paralysis, tetrodotoxin poisoning, coonhound paralysis, or vestibular disease). Caution should also be taken in extrapolating the proposed case definition to regions outside of the Pacific. All of the data on which the case definition is based comes from CFP cases in countries surrounded by or bordering the Pacific Ocean. However, the symptomology of human CFP is known to vary between the Pacific Ocean, the Indian Ocean, and the Caribbean [23]. Finally, the timing of case classification is important. The proposed case definition is less sensitive when applied on presentation (Table-12); therefore, it is recommended to reassess case classification as additional clinical signs emerge.

## Conclusion

This case series is the largest study of canine and feline CFP to date. It documented a multisystem toxicosis characterized primarily by motor dysfunction. Evidence was found of differences between canine and feline CFP, and the results also document previously undescribed clinical signs and events.

The findings from this study, along with the existing literature, informed the development of the first-ever case definition (and diagnostic algorithm) for CFP in dogs and cats. This diagnostic tool will assist clinicians in determining the probability of CFP in patients and will provide researchers with a means of distinguishing cases from non-cases. The proposed case definition is not touted as a perfect solution. Rather, it is a suggestion that will need refinement as more is learned about CFP in dogs and cats.

The limitations of this study highlight the need for further research. One key issue is that the syndrome described was only indirectly attributed to CFP. A prospective study with laboratory detection of CTX is needed. Given that fish ingestion was infrequently observed in this case series, the development of an assay procedure for biologic samples is recommended. Additional descriptive studies of CFP cases originating in the Indian Ocean and the Caribbean are also recommended to establish whether the clinical presentation of canine and feline CFP differs between regions.

## Author’s Contributions

MJG designed the study, collected the data, and wrote the manuscript. MCG performed the statistical tests and contributed the associated methods. Both authors read and approved the final manuscript.

## Supplementary Tables

**Supplementary Table-1:** Variables of interest.

Case Exposure	Known/unknown fish ingestion
	Date of fish ingestion
	Type of fish ingested
	Source of fish
	Date of onset of clinical signs
Neurologic Signs^1^	Ataxia
	Proprioceptive deficits
	Paresis/paralysis/weakness
	Hindlimbs worse than forelimbs
	Recumbency/inability to walk
	Hypertonus/extensor rigidity
	Opisthotonos
	Convulsions/seizures
	Tremors
	Hypaesthesia/dysaesthesia
	Nystagmus
	Loss of gag reflex
	Mydriasis
	Lacrimation
	Lethargy
	Obtunded mentation
	Vocalisation
Cardiovascular and Respiratory Signs^1^	Dehydration
	Dyspnoea (severity)
	Cardiac irregularities
	Groaning
Gastrointestinal Signs^1^	Anorexia/Inappetence
	Abdominal discomfort
	Diarrhoea
	Salivation
	Vomiting

1 Clinic signs were selected based upon those reported in previous publications.

**Supplementary Table-2:** Clinical signs of canine CFP: comparison of cases with documented fish ingestion versus those with unknown exposure history.

Clinical Sign	Frequency in dogs with documented fish ingestion (n=48)	Frequency in dogs with unknown exposure history (n=113)	p value (Fisher’s exact test)	Odds ratio associated with significant p values (min-max)
Ataxia	68.8%	62.8%	0.38	
Inappetence/anorexia	54.2%	57.5%	1	
Recumbency	52.1%	67.3%	0.16	
Tachypnoea/dyspnoea	52.1%	54.9%	1	
Hypertonus/extensor rigidity	47.9%	49.6%	1	
Unable to walk	41.7%	26.5%	0.06	
Paresis/paralysis/weakness	37.5%	53.1%	0.12	
Groaning	33.3%	37.2%	0.86	
Obtunded mentation	31.3%	24.8%	0.34	
Vocalisation	31.3%	26.5%	0.56	
Diarrhoea	25.0%	10.6%	0.026	2.9 (1.1 -7.8)
Hypersalivation	25.0%	18.6%	0.39	
Opisthotonos	22.9%	30.1%	0.45	
Nystagmus	20.8%	19.5%	0.83	
Cardiac irregularities	18.8%	7.1%	0.044	3.1 (1.0- 10.0)
Tremors	18.8%	23.9%	0.68	
Lethargy	16.7%	15.0%	0.81	
Hindlimbs worse than forelimbs	14.6%	29.2%	0.073	
Vomiting	14.6%	15.0%	1	
Dehydration	12.5%	11.5%	0.79	
Proprioceptive deficits	12.5%	17.7%	0.64	
Convulsions/seizures	8.3%	7.1%	0.75	
No gag reflex	8.3%	8.8%	1	
Hyperaesthesia/dysaesthesia	6.3%	4.4%	0.69	
Abdominal discomfort	0.0%	13.3%	0.006	0 (0- 0.6)

**Supplementary Table-3:** Clinical signs of feline CFP: comparison of cases with documented fish ingestion versus those with unknown exposure history.

Clinical Signs	Frequency in cats with documented fish exposure (n=22)	Frequency in cats with unknown exposure history (n=55)	p value (Fisher’s exact test)
Ataxia	86.4%	74.5%	0.40
Inappetence/anorexia	63.6%	50.9%	0.33
Recumbency	59.1%	60.0%	1
Paresis/paralysis/weakness	45.5%	50.9%	0.81
Tremors	45.5%	23.6%	0.10
Dehydration	36.4%	34.5%	1
Hindlimbs worse than forelimbs	36.4%	32.7%	0.79
Hypertonus/extensor rigidity	31.8%	47.3%	0.32
Obtunded mentation	27.3%	25.5%	1
Opisthotonos	27.3%	25.5%	1
Proprioceptive deficits	27.3%	21.8%	0.77
Tachypnoea/dyspnoea	27.3%	29.1%	1
Unable to walk	27.3%	41.8%	0.31
Hypersalivation	22.7%	10.9%	0.28
Groaning	13.6%	1.8%	0.067
Hyperaesthesia/dysaesthesia	13.6%	16.4%	0.75
Lethargy	13.6%	10.9%	1
Nystagmus	13.6%	29.1%	0.25
Cardiac irregularities	9.1%	7.3%	1
Diarrhoea	9.1%	1.8%	0.19
Vocalisation	9.1%	23.6%	0.14
Vomiting	9.1%	1.8%	0.19
No gag reflex	4.5%	1.8%	0.49
Abdominal discomfort	0.0%	1.8%	1
Convulsions/seizures	0.0%	12.7%	0.18

**Supplementary Table-4:** Clinical signs of CFP: comparison of canine and feline cases.

Clinical sign	Frequency in dogs (n=161)	Frequency in cats (n=77)	p value (Fisher’s exact test)	Odds ratio associated with significant p values (min-max)
Ataxia	64.60%	77.90%	0.11	
Recumbency	62.70%	59.70%	0.58	
Inappetence/anorexia	56.50%	54.50%	0.68	
Tachypnoea/dyspnoea	54.00%	28.60%	0.0002	0.3 (0.2- 0.6)
Hypertonus/extensor rigidity	49.10%	42.90%	0.34	
Paresis/paralysis/weakness	48.40%	49.40%	1	
Groaning	36.00%	5.20%	0.00000004	0.1 (0.0- 0.3)
Unable to walk	31.10%	37.70%	0.39	
Vocalisation	28.00%	19.50%	0.21	
Opisthotonos	28.00%	26.00%	0.76	
Obtunded mentation	26.70%	26.00%	0.88	
Hindlimbs worse than forelimbs	24.80%	33.80%	0.22	
Tremors	22.40%	29.90%	0.27	
Hypersalivation	20.50%	14.30%	0.29	
Nystagmus	19.90%	24.70%	0.50	
Proprioceptive deficits	16.10%	23.40%	0.16	
Lethargy	15.50%	11.70%	0.70	
Vomiting	14.90%	3.90%	0.009	0.23 (0.0- 0.8)
Diarrhoea	14.90%	3.90%	0.009	0.23 (0.0- 0.8)
Dehydration	11.80%	35.10%	0.00009	3.8 (1.9- 7.9)
Cardiac irregularities	10.60%	7.80%	0.64	
Abdominal discomfort	9.30%	1.30%	0.024	0.1 (0.0-0.8)
No gag reflex	8.70%	2.60%	0.10	
Convulsions/seizures	7.50%	9.10%	0.80	
Hyperaesthesia/dysaesthesia	5.00%	15.60%	0.003	4.1 (1.5- 11.8)

**Supplementary Table-5:** Clinical signs of CFP: signs manifest on presentation versus overall frequency.

Clinical sign	Number of animals affected on presentation^1^/number affected in total (%)	Number of dogs affected on presentation^1^/number affected in total (%)	Number of cats affected on presentation^1^/number affected in total (%)
Convulsions/seizures	5/19 (26%)	3/12 (25%)	2/7 (29%)
Opisthotonos	20/65 (31%)	13/45 (29%)	7/20 (35%)
Nystagmus	19/51 (37%)	10/32 (31%)	9/19 (47%)
No gag reflex	7/16 (44%)	5/14 (36%)	2/2 (100%)
Diarrhoea	12/27 (44%)	10/24 (42%)	2/3 (67%)
Vocalisation	28/60 (47%)	18/45 (40%)	10/15 (67%)
Tremors	29/59 (49%)	16/36 (44%)	13/23 (57%)
Inappetence/anorexia	66/133 (50%)	40/91 (44%)	26/42 (62%)
Recumbency	79/147 (54%)	55/101 (54%)	24/46 (52%)
Obtunded mentation	35/63 (56%)	25/43 (58%)	10/20 (50%)
Hypertonus/extensor rigidity	65/112 (58%)	44/79 (56%)	21/33 (64%)
Vomiting	16/27 (59%)	15/24 (63%)	1/3 (33%)
Ataxia	104/164 (63%)	62/104 (60%)	42/60 (70%)
Paresis/paralysis/weakness	74/116 (64%)	46/78 (59%)	28/38 (74%)
Groaning	40/62 (65%)	37/58 (64%)	3/4 (75%)
Hyperaesthesia/dysaesthesia	13/20 (65%)	5/8 (63%)	8/12 (67%)
Hypersalivation	29/44 (66%)	22/33 (67%)	7/11 (64%)
Unable to walk	56/79 (71%)	35/50 (70%)	21/29 (72%)
Lethargy	25/34 (74%)	19/25 (76%)	6/9 (67%)
Dehydration	35/46 (76%)	15/19 (79%)	20/27 (74%)
Proprioceptive deficits	34/44 (77%)	21/26 (81%)	13/18 (72%)
Hindlimbs worse than forelimbs	52/66 (79%)	31/40 (78%)	21/26 (81%)
Tachypnoea/Dyspnoea	89/109 (82%)	74/87 (85%)	15/22 (68%)
Cardiac irregularities	19/23 (83%)	15/17 (88%)	4/6 (67%)
Abdominal discomfort	16/16 (100%)	15/15 (100%)	1/1 (100%)

1 Clinical sign documented on the first day of hospitalisation
